# Onycophagie

**Published:** 1894-03

**Authors:** 


ARTICLE II.

### "ONYCOPHAGIE."

NAIL-BITING AMONG CHILDREN AND THE CAUSE OF IT.—FRENCH EXPERIMENT IN A NEW FIELD.—INVESTIGATION OF THE PRACTICE LEADS TO THE CONCLUSION THAT IT IS AN UNFAILING MARK OF INCIPIENT DEGENERATION OF THE NERVOUS SYSTEM.

There has just been issued from the Paris press a *brochure* which is creating a large amount of interest in French medical circles, both on account of its originality and the experimental results which it embodies. It is from the pen of Dr. Berillon, so well known in the surgical world by reason of his prominent connection with the dead Charcot in the latter's hypnotic experiments, and at present secretary-general de la Societe d'Hypnologie et de Psychologie of Paris and medical inspector of the State lunatic asylums.

The work is a scientific treatise on onychophagie, or finger-nail biting, and contains the results of a series of observations in the public and private schools of France, and extending through a period of more than seven years. At the congress of the French Association for the Advancement ot Sciences, held at Nancy, in 1886, Berillon first announced his observations on the habit of nail-biting, and since that time has been in almost continuous experiment.

In his thoroughly scientific treatment of what the world has never before eonsidered worthy of prolonged or special study, Berillon has arrived at results really remarkable. His experiments lead him to pronounce the habit far more widespread and pernicious than others promptly treated, and force him to conclude that if not a disease itself, it is an unfailing mark of incipient degeneration of the nervous system, which, recognized, must be treated promptly, and, unrecognized, may be productive of the most evil results.

The following will show the nature of the observations made in the schools:

In a mixed school of the department of l'Yonne the reports showed the following results:

| | NUMBER EXAMINED. | NAIL-BITERS. |
|---|---|---|
| Boys, - | 29 | 6 |
| Girls, - - | 21 | 11 |
| Total, | 50 | 17 |

The proportion of nail-biters here was for boys 20 per cent. and for girls 52 per cent.

In a boys' school at Seine-et-Marne the pupils were examined with respect to age with the following results:

| | NUMBER EXAMINED. | NAIL-BITERS. |
|---|---|---|
| 12 to 14 years, | 18 | 7 |
| 13 to 15 years, | 16 | 6 |
| 15 to 17 years, | 18 | 3 |
| Total, | 52 | 16 |

From twelve to fourteen seems from this to be the age more susceptible to this habit. A like experiment with girls shows them to be even more susceptible at this age.

| | NUMBER EXAMINED. | NAIL-BITERS. |
|---|---|---|
| 10 to 13 years, | 80 | 27 |
| 12 to 15 years, | 75 | 21 |
| 15 to 16 years, | 52 | 13 |
| 16 to 17 years, | 10 | 2 |
| Total, | 217 | 63 |

In all the schools where the children have been the objects of careful and attentive observation, the reports have agreed in pronouncing that pupils observed to have the habit are universally the poorest students; that if boys they are inclined to effeminacy and if girls to slackness. In many there are marked defects of character and less sustained attention. The reports of writing-masters, also, declare their writing to be, while sometimes well-formed, universally less legible and less regular, and the instructors in the Parisian schools for manual training have pronounced the habitual nail-biters hardest of all to teach and often unfit for technical education.

Among such pupils some have shown brilliant intellectual traits. Some are possessed of an astonishing memory or show exceptional adaptability to certain arts or certain special studies. Of these "infant prodigies" a large proportion are found to be nail-biters. In such cases the exceptional brilliancy was of unnatural and ephemeral growth and vanished at the age of fourteen or fifteen. The extraordinary development had compromised the normal evolution of the nervous system.

In schools for children from six to eight years old those pupils cited by the matrons as most incorrigible and upon whom fell the most constant discipline were found, almost without exception, to be possessed of the habit.

In general, the nail-biters were found to be of decided inferiority, both from a point of view of intellectual development and from that of moral sensibility.

Having thus convinced himself of the extreme commonness of the nail-biting habit, and finding it constantly associated with evil characteristics in the child, Berillon started an exhaustive series of observations upon its nature and consequence.

Fonssagrives, who in his "Dictionnaire de la Sante" was the first to recognize the pernicious effects of nail-biting in the young, referred it to the instinct of the infant, which impels it to carry all objects with which it comes in contact to its mouth. The habit of sucking the thumb or fingers, he argued, was merely the continuation, by simple habit, of an impulse primitive and instinctive, and the after transformation of this impulse into an act automatic and unconscious. Cases are known, it is true, where this habit has lasted even till maturity, when it had become a mania, resulting in complete deformation of the mouth and the fingers. In most cases, however, the habit lingers with the child in the form of nail-biting.

Berillon's experiments have carried him further than did Fonssagrives's. He finds the number of children who have acquired the habit only after arriving at a reasoning age to be fully as great, if not greater, than those in whom the habit has been observed since infancy. Its origin in them, therefore, must be explained by other hypotheses than those of continuation of primitive impulses.

In many cases the sudden appearance of the habit may be clearly traced to imitation. Berillon finds many cases of children from seven to ten years of age who have never had the habit, but placed in a new school have been observed at the end of a month to have contracted it. In every case several of the child's comrades were nail-biters, and the one contracting the habit was delicate and easily influenced.

There has remained, however, in all his experiments a large proportion of cases which show a late contraction of the habit and one which cannot be traced to imitation. It is to those cases that most of Berillon's study has been directed.

Most curiously, he has established the fact that nail-biting is in most cases hereditary. This conclusion was reached after an inquiry into the antecedents of hundreds of cases examined. It is extremely rare, he says, to find a child in whom the habit is pronounced whose antecedents, direct or collateral, have not been themselves nail-biters.

This odd fact was of such constant recurrence that it led him to a more careful study of the conditions of his cases, and brought him to the conviction that nail-biting is, in almost all cases, not to be looked at as merely a child's habit, unpleasant and punishable, but as an indication of some hereditary physical or mental degeneration.

In such families the hereditary degeneration is to be observed, unhappily, in more than mere nail-biting. Often the heads of such children present species of deformations, such as microcephalis, (small head,) bony crests, or protuberances on different parts of the head, while the face reveals crossed eyes, nearsightedness, irregular teeth or displacement of features.

At this point, revealing his inquiry, Berillon gave his attention to those thus afflicted in the Paris hospital and found that a very large proportion of the children of these showed the habit of nail-biting, with other signs of physical degeneration.

Berillon's experiments prove, then, that nail-biting, as a habit in children, has its source deeper than mere imitation of childish idiosyncrasy. He shows it to be no willful habit, to be cured by ordinary petty punishment, but an indication of an incipient nervous degeneration, which, once observed, may be treated understandingly in the very beginning. The outward habit, if it do not disappear when the primary cause be removed, will yield to ordinary treatment.

Berillon, then, by a series of experiments in which he isolates the habit of nail-biting from other signs of degeneration, seeks to determine the reflex effect of the habit upon the nervous system of the child in whom it is allowed to exist.

One of the most remarkable properties of the nervous system is a tendency to automatic activity. The performance of an act and its repetition increases the tendency to do it again, and in time, if yielded to, it grows irresistible. This act is often unconscious, as is proved by the fact that it is more frequent when the attention is engaged, and there is no will to resist the automatic action. "Thus, children," says Berillon, "bite the nails while learning their lessons, or even while asleep." The tendency to automatic repetition has long been recognized to be an indication of physical degeneration.

Upon this ground a long series of experiments upon men whose occupation or employment had been such as to consist, in great part, of such automatic action and the constant existence in them of physical degeneration points to the fact that this degeneration is increased and hastened to a great degree by this automatic repetition. This is true, however, of all nervous diseases. Hysteria, for example, increases in exact proportion to the number of *crises* or paroxysms of the patient, and when these are decreased in number by mere effort of the patient's will or by hypnotic suggestion—the method now employed in the French hospitals—the disease decreases in the same measure.

It follows, then, that the nail-biting habit, so far from being harmless in the child, is most pernicious and productive of most evil results upon the nervous system, and through that upon the general health of the child.

Not only in France has been recognized the commonness and the evil effects of the habit. In English schools it has been noted in a great number of cases. Instances are cited where, in classes of thirty children of parents in good circumstances, an average of 50 per cent. were observed to be nail-biters. In England, indeed, the habit is considered so harmful that in certain schools the hands of the pupils are the objects of frequent inspection and the nail-biters are severely and publicly reprimanded. But always, in England as well as in France, such punishments do not seem to have the effect of curing the habit or decreasing the proportion of those addicted to it.

In Paris the usual treatment has been to rub the extremities of the fingers with aloes, bitter almonds, sulphate of quinine, macerated quassia, etc. Some physicians counsel the constant wearing of gloves or advise putting them on at night. But the insufficiency of all these means has long been demonstrated. The child takes up the vicious habit as soon as the means of coercion are removed.

As to the cure of this peculiar disease Berillon has much to say. He advocates strongly the grouping in classes of children of the *Lysees* who manifest the habit, where they may be subjected to special discipline and hygienic treatment on the order of that which is employed in the English system of reformatory schools. He is opposed to the isolation of pupils who have the habit, as practiced in England, on the ground that cure by this means is only transient. Regarding the habit as an indication of nervous degeneration, he would, in all cases, begin by a careful system of food and exercise, and thus try by every means to fortify the nervous system of the child. For curing the after-habit, which continues when the cause has been removed, he employs his own treatment, which is as follows: 1. To create a counter-irritant and thus to transform by outward excitation the unconscious act into a conscious one. 2. To create a counter-automatic impulse. 3. To strengthen the resolution of the child. He adds to these the treatment by suggestion while in hypnotic sleep.

In pursuance of his observations on this subject, blank forms have been sent out, and by this means there has been begun a more complete examination of all the schools of France, so that under his direction there can be made a most careful study of the newly diagnosed disease.—*Baltimore Sun.*

